# Prognostic impact of platinum sensitivity in ovarian carcinoma patients with brain metastasis

**DOI:** 10.1186/s12885-019-6382-x

**Published:** 2019-12-05

**Authors:** Alexandre André Balieiro Anastácio da Costa, Elizabeth Santana dos Santos, Deborah Porto Cotrim, Natasha Carvalho Pandolfi, Marcelle Goldner Cesca, Henrique Mantoan, Solange Moraes Sanches, Adriana Regina Gonçalves Ribeiro, Louise de Brot, Graziele Bonvolim, Paulo Issamu Sanematsu, Ronaldo Pereira de Souza, Joyce Maria Lisboa Maya, Fabrício de Souza Castro, João Paulo da Nogueira Silveira Lima, Michael Jenwel Chen, Andrea Paiva Gadelha Guimarães, Glauco Baiocchi

**Affiliations:** 1 Medical Oncology Department, A.C. Camargo Cancer Center, 211 Professor Antonio Prudente Street, Liberdade, São Paulo, SP 01509-900 Brazil; 2 Gynecology Oncology Department, A.C. Camargo Cancer Center, 211 Professor Antonio Prudente Street, Liberdade, São Paulo, SP 01509-900 Brazil; 3 Pathology Department, A.C. Camargo Cancer Center, 211 Professor Antonio Prudente Street, Liberdade, São Paulo, SP 01509-900 Brazil; 4 Neurosurgery Department, A.C. Camargo Cancer Center, 211 Professor Antonio Prudente Street, Liberdade, São Paulo, SP 01509-900 Brazil; 5 Radiotherapy Department, A.C. Camargo Cancer Center, 211 Professor Antonio Prudente Street, Liberdade, São Paulo, SP 01509-900 Brazil

**Keywords:** Brain metastasis, Prognostic factors, Ovarian cancer, Platinum sensitivity

## Abstract

**Background:**

Brain metastasis (BM) is a rare event in ovarian cancer patients. The current prognostic scores that have been used for other tumors do not account for specific characteristics of ovarian cancer, such as platinum sensitivity.

**Methods:**

This retrospective cohort study examined patients with ovarian carcinoma and BM who were treated at a single institution from January 2007 to December 2017. Clinical data on the diagnosis of BM and follow-up were collected. Cox regression was used to evaluate prognostic factors for overall survival (OS).

**Results:**

Of 560 patients, 26 presented with BM. Eight patients were treated with surgery, 15 with whole-brain radiotherapy (RT), and 5 with stereotactic RT, and 4 patients received systemic treatment at the diagnosis of BM. The median OS was 10.8 months. The following factors were associated with OS: platinum-sensitive recurrence (HR 0.34, 95% CI 0.12–0.99; *p* = 0.049), higher number of previous treatment lines (HR 1.57, 95% CI 1.12–2.19; *p* = 0.008), ECOG performance status (HR 2.52, 95% CI 1.24–5.09; *p* = 0.010), and longer interval from initial diagnosis to BM (*p* = 0.025). Notably, the number of brain metastasis, the largest tumor size, and progression outside of the CNS were not related to survival. Platinum sensitivity was not associated with any of the classic prognostic factors in brain metastasis patients such as number or size of brain metastasis or disease progression outside the CNS strengthening the hypothesis of the importance of platinum sensitivity to the prognosis of ovarian cancer patients with BM.

**Conclusions:**

The factors related to the biological behavior of the ovarian cancer such as platinum sensitivity at the time of BM diagnosis, fewer number of previous treatment lines and interval from initial diagnosis were associated with survival in ovarian cancer patients with BM, while factors that are usually related to survival in BM in other cancers were not associated with survival in this cohort of ovarian cancer patients. The small number of patients did not allow us to exclude the prognostic role of these former factors that were not associated with survival in the present cohort.

## Background

Ovarian cancer is the eighth most frequent cancer among women, amounting to 295,414 cases worldwide in 2018 [1]. It is the most lethal gynecological cancer, accounting for 2.5% of female malignancies but 5.0% of deaths due to cancer in women [2]. Brain metastasis (BM) is an uncommon event in ovarian cancer patients and is estimated to occur in 0.3 to 12% of patients, depending on the series. The highest frequencies have been seen in more recent series, likely due to the advent of more accurate diagnostic tools and longer survival in this era of new treatment options [3].

BM can be treated with various combinations of surgery, whole-brain radiotherapy, and stereotactic radiotherapy. Combination of surgery and radiotherapy yields better survival than single modality treatment alone, and the prognosis is a key component of the treatment decision, surgery usually reserved for patients with 1 to 3 lesions, controlled systemic disease and good performance status [4]. The response to systemic treatment including chemotherapy or targeted therapy in the BM seems to parallel the response of the primary tumor [4].

Classical prognostic scores as the RTOG Recursive Partitioning Analysis (RPA), and the grade prognostic index (GPA) have mainly accounted for primary tumors with the highest frequencies of BM, such as melanoma and lung, breast, and kidney cancer [5, 6]. Aiming to refine the prognostic evaluation for specific primary tumor sites the Disease-Specific Grade Prognostic Index (DS-GPA) was developed, for which only melanoma, lung, breast, kidney, and gastrointestinal cancer have been studied [7]. In the classical non-disease specific scores and in the scores for most studied primary sites, age, KPS, extracranial metastases, and the number of BMs are relevant prognostic factors [5–7].

Due to its rarity, BM from ovarian carcinoma is underrepresented in these studies. BM from ovarian carcinoma has been lumped with other primary gynecological tumors [8–12] and in small series of ovarian carcinoma cases [13–18]. Only 2 recent series presented more than 40 ovarian carcinoma patients with BM [13, 15]. In these series the prognostic factors most often associated with survival were performance status, size and number of brain metastasis [8–18]. Only three of these series evaluated platinum sensitivity at the time of BM diagnosis as prognostic factors for survival [13, 15, 16].

In this study, we evaluated prognostic factors in a cohort of ovarian cancer patients who presented with BM, considering the specific characteristics of ovarian cancer treatment and its natural history.

## Methods

### Patients

This retrospective cohort study comprised all consecutive patients with ovarian carcinoma who were treated at A.C. Camargo Cancer Center from January 2007 to December 2017, irrespective of the date of diagnosis, and who developed brain metastasis (BM).

The study was conducted in accordance with the Declaration of Helsinki ethical guidelines and approved by the institutional Ethics Committee (CEP# 2649/18).

### Clinical data

Clinical findings were retrieved from the medical records. Baseline characteristics included the date of diagnosis, age at diagnosis of ovarian cancer, tumor histological subtype, staging, residual disease after debulking surgery, and personal history of ovarian and breast cancer. Clinical characteristics at the time of the diagnosis of BM included: type of recurrence regarding platinum sensitivity, number of previous chemotherapy treatment lines, sites of metastatic disease, status of extra-nervous system disease progression, number of BMs, size of largest BM, platinum-free interval, and time from diagnosis. Data on treatment modality at the time of BM were also retrieved.

Recurrence was defined per the GCIG (Gynecological Cancer Intergroup) criteria for CA125 progression or per RECIST (Response Evaluation Criteria in Solid Tumors) for image studies obtained from the medical records. The date of the earliest event was considered for progression [19, 20]. Recurrence that was detected after 6 months of the last platinum infusion was defined as platinum-sensitive recurrence, whereas that within 6 months was considered platinum-resistant recurrence. All recurrences that followed the initial platinum-resistant recurrence were also considered to be platinum-resistant. Overall survival was defined as the interval between the date of the diagnosis of BM and death due to any cause. The interval between the date of the last platinum compound infusion and the date of the diagnosis of BM was defined as the platinum-free interval (PFI).

### Statistical analysis

Frequencies, medians, and interquartile range (IQR) were used to describe the patients’ characteristics. The association between clinical characteristics was tested by Qui-square Test or Fisher’s Exact test when necessary. Overall survival curves were plotted by Kaplan-Meier method and we used log-rank test to test the association between clinical characteristics and overall survival. Hazard ratios were calculated by Cox regression analysis, but due to the small number of patients, only univariate analysis was performed.

When necessary, continuous variables were dichotomized using the medium value of the cohort as the cutoff. The statistical analysis was performed using SPSS v. 21.0 (SPSS, Chicago, IL, US), adopting a two-tailed *p* value < 0.05 as significant.

## Results

Of the 560 patients with ovarian carcinoma treated at A.C. Camargo Cancer Center from January 2007 to December 2017, 26 (4.6%) developed BM during the follow-up. The median age at the diagnosis of BM was 63.0 years, and the median time from the primary diagnosis to BM was 31.7 months; most patients presented with high-grade serous carcinoma and had a negative family history for breast or ovarian cancer and an ECOG performance status of 0 or 1 (Table 1).

**Table 1:** Clinical characteristics of the 26 ovarian cancer patients with brain metastasis

Characteristic	Freq. (%)
Age (median / IQR)	63.0 (54.1–65.7)
Family history	
Present	5 (19.2)
Absent	19 (73.1)
Unknown	2 (7.7)
Histology	
High grade serous	17 (65.4)
Endometrioid	2 (7.7)
Undifferentiated	2 (7.7)
Carcinossarcoma	1 (3.8)
Mixed	1 (3.8)
Unknown	3 (11.5)
FIGO stage	
I-III	20 (76.9)
IV	5 (19.2)
Unknown	1 (3.8)
ECOG performance status	
0	4(15.4)
1	12 (46.2)
2	2 (7.7)
3	3 (11.5)
Unknown	5 (19.2)
Residue disease after first surgery	
> 1 cm	18 (69.2)
< 1 cm	5 (19.2)
Unknown	3 (11.5)

*IQR* Interquartile range, *FIGO* International Federation of Gynecology and Obstetrics, *ECOG* Eastern Cooperative Oncology Group

Approximately 50% of the patients experienced a recurrence that was classified as platinum-sensitive, 46.2% was treated with just one previous line of chemotherapy, and 57.4% did not have concurrent disease progression outside of the central nervous system, and in 6 (23.2%) of these patients BM was the only site of metastatic disease. The number of BMs varied widely, and one-third of patients presented with a single BM, compared with one-fourth who developed multiple (> 20) BMs. The median size of the BMs was 3.2 cm. Roughly 80% of patients were treated with radiotherapy; 8 patients (20.8%) underwent surgery, all of whom were administered whole-brain radiotherapy or stereotaxic radiotherapy afterward (Table 2).

**Table 2:** Characteristics of CNS metastasis

Characteristic	Freq. (%)
Time-point of CNS metastasis	
Platinum sensitive	14 (53.8)
Platinum resistant	11 (42.3)
Unknown	1 (3.8)
Previous CT lines before diagnosis CNS metastasis	
1	12 (46.2)
2	2 (7.7)
3	3 (11.5)
4	5 (19.2)
5	3 (11.5)
Unknown	1 (3.8)
BM as only site of metastatic disease	
Yes	6 (23.2)
No	20 (76.9)
Extra-CNS disease progression	
Yes	11 (42.6)
No	14 (53.8)
Unknown	1 (3.8)
Number of metastasis (median /IQR)	4.5 (1–20)
Metastasis size (median/IQR)	3.2 (2.1–3.7)
Platinum free interval (median/IQR)	10.9 (4.2–22.4)
Time from diagnosis (median/IQR)	31.8 (18.9–55.8)
Treatment	
Surgery + WBRT or SRT	8 (30.8)
WBRT	15 (57.7)
SRT	5 (19.2)
Chemotheraphy	4 (15.4)

*CNS* Central nervous system, *CT* Chemotherapy, *IQR* Interquartile range, *WBRT* Whole brain radiotherapy, *SRT* Stereotaxic radiotherapy

### Overall survival

**Fig. 1:**
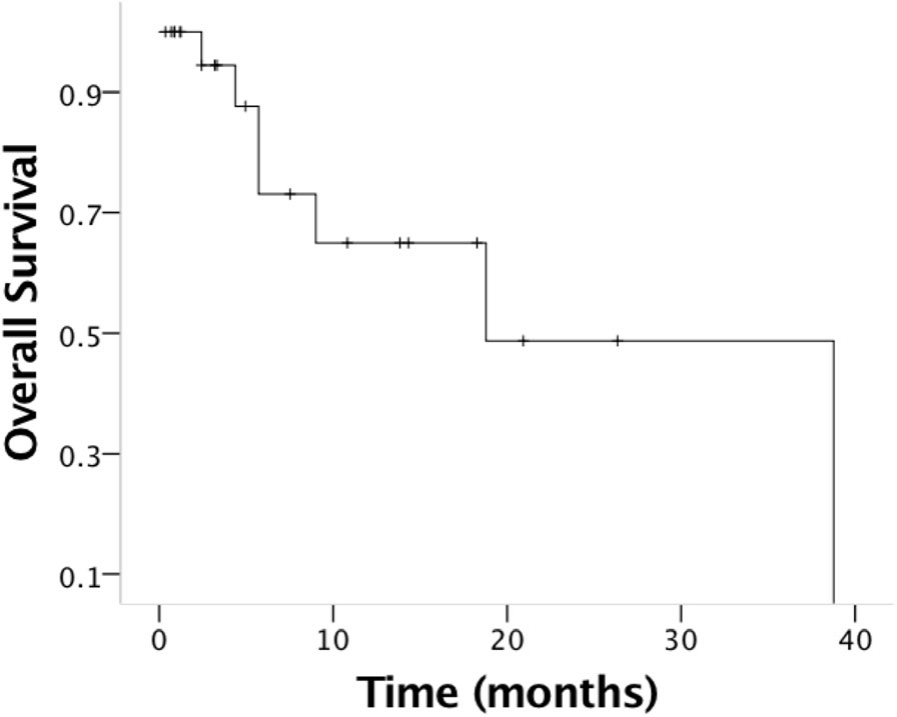
Overall survival for the 26 patients with brain metastasis from ovarian carcinoma. The median overall survival was 18.8 months

**Fig. 2:**
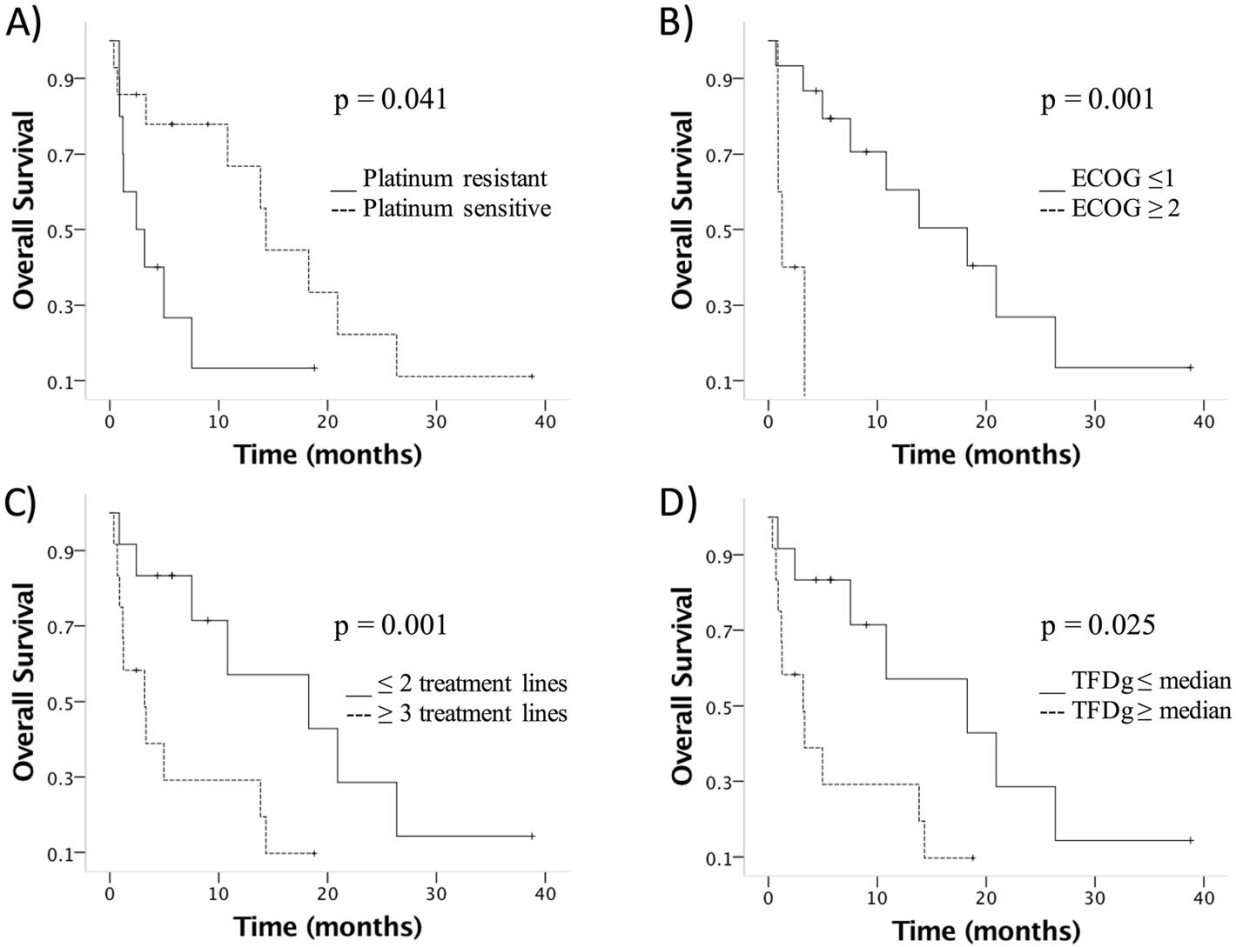
Overall survival according to clinical characteristics. **a** Platinum sensitivity, **b** performance status, **c** number of previous treatment lines, and **d** time from primary diagnosis to the diagnosis of brain metastasis (TFDg). *p* values calculated by Log Rank test

At a median follow-up of 18.7 months since the diagnosis of BM, the median overall survival was 10.8 months (Fig. 1). Of the 18 patients who died, the cause of death was related to BM in 13 patients (72.2%). For the other 5 patients who died the cause of death was: bowel obstruction due to peritoneal carcinomatosis in two patients, respiratory failure due to lung metastasis in one patient and pleural metastasis in another patient, and cholangitis due to liver metastasis in one patient. The following factors were associated with overall survival: platinum-sensitive recurrence (HR 0.34, 9y5% CI 0.12–0.99; *p* = 0.049), higher number of previous treatment lines (HR 1.57, 95% CI 1.12–2.19; *p* = 0.008), and ECOG performance status (HR 2.52, 95% CI 1.24–5.09; *p* = 0.010) (Fig. 2 and Table 3).

**Table 3:** Univariate analysis for Overall Survival

Characteristic	HR (95% CI)	*p* value
Age^a^	1.02 (0.96–1.08)	0.493
ECOG performance status^a^	2.52 (1.25–5.09)	0.010
Family History of Breast or Ovarian Cancer		
No	1	0.593
Yes	0.73 (0.23–2.30)	
Histology		
HGCS	1	0.747
Other histologies	0.81 (0.22–3.00)	
Time-point of CNS metastasis		
Platinum sensitive	1	0.049
Platinum resistant	2.89 (1.01–8.31)	
Previous lines before diagnosis of BM^a^	1.57 (1.12–2.19)	0.008
Disease progression outside CNS		
No	1	0.236
Yes	0.52 (0.18–1.53)	
Number of metastasis^a^	1.06 (0.99–1.13)	0.104
Metastasis size^a^	0.83 (0.49–1.39)	0.473
Time from diagnosis^a^	1.01 (1.00–1.01)	0.200
Platinum free interval^a^	0.99 (0.93–1.05)	0.747

*ECOG* Eastern Cooperative Oncology Group, *HGSC* High grade serous carcinoma, *CNS* Central nervous system

^**a**^evaluated as continuous variables

**Fig. 3:**
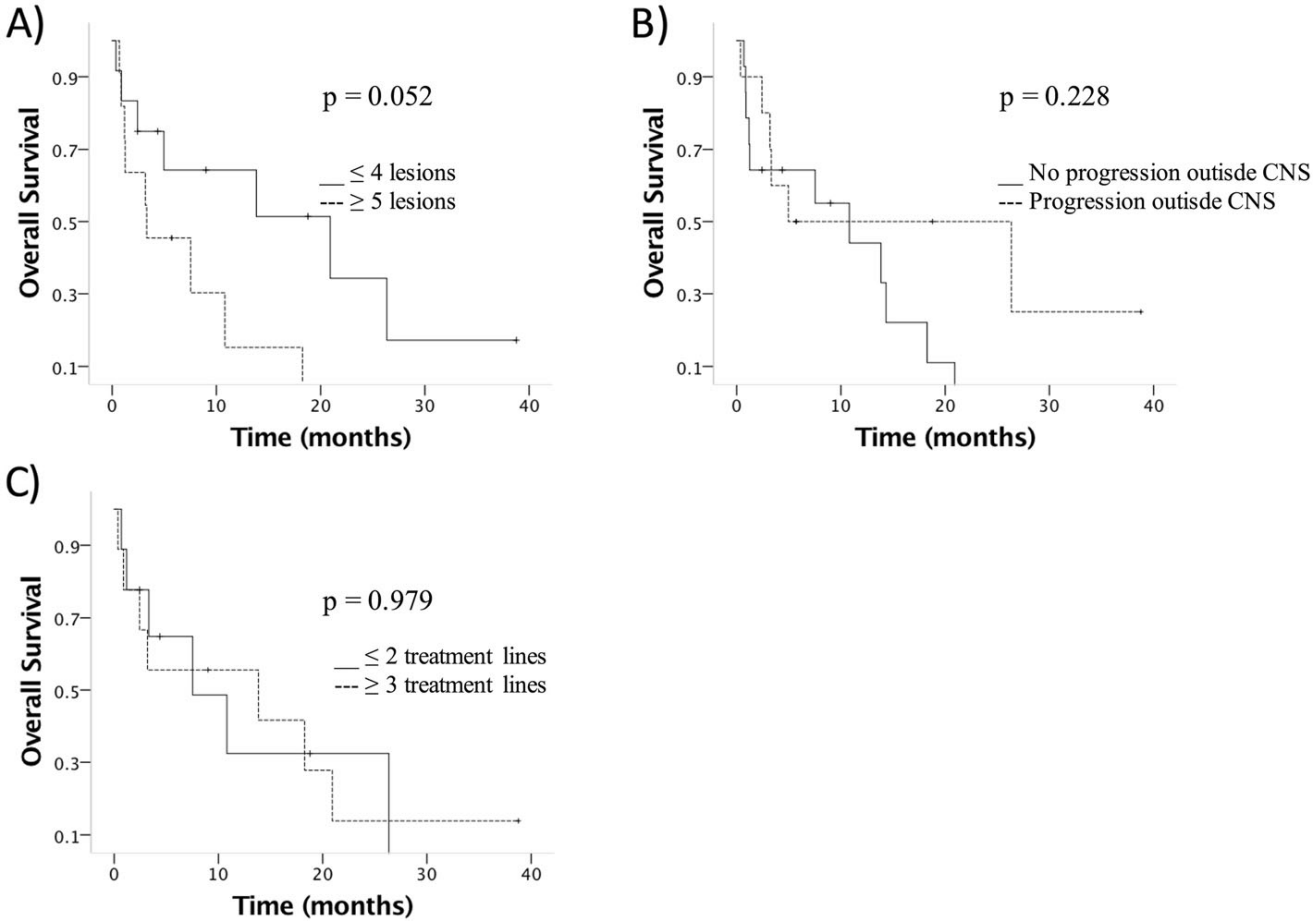
Overall survival according to clinical characteristics. **a** Number of metastatic lesions to the brain, **b** disease progression outside of the CNS, and **c** size larger than 3.2 cm (median for the cohort). p values calculated by Log Rank test

Using median values as cutoffs for continuous variables, the time from diagnosis was associated with overall survival (HR 3.27, 95% CI 1.10–9.71, *p* = 0.033), and patients with 4 or fewer BMs had better overall survival compared with those with 5 or more BMs (HR 2.91, 95% CI 0.95–8.99; *p* = 0.063). Median tumor size larger than 3.2 cm (HR 1.02, 95% CI 0.338–3.05, *p* = 0.979), and progression outside of the CNS (HR 0.52, 95% CI 0.18–1.53, *p* = 0.236) were not associated with survival (Fig. 3 and Table 3).

### Association of platinum sensitivity and characteristics of brain metastasis

In order to evaluate if the association of platinum sensitivity with survival was due to a confounder effect of its association with the classic prognostic factors, we evaluated the association of platinum sensitivity with other clinical characteristics.

Residual disease after the initial surgery (primary debulking or interval debulking surgery) that exceeded 1 cm was related to the diagnosis of BM in the platinum-resistant setting. All 5 patients with residual disease > 1 cm had a diagnosis of BM in the platinum-resistant setting (*p* = 0.014). A diagnosis of BM after 3 or more chemotherapy treatment lines was also associated with platinum resistance, 11 of 14 patients with less than 3 previous treatment lines had platinum sensitive recurrence while 3 of 11 patients with more than 3 previous treatment lines had platinum resistant recurrence at the time of diagnosis of BM (*p* = 0.017).

Notably, there was no association between a diagnosis of BM in the platinum-resistant setting and having 1 versus > 1 metastasis (*p* = 0.423), BM larger than 3.2 cm (median for the cohort) (*p* = 0.650), or concurrent disease progression outside of the central nervous system (*p* = 1.00). Moreover, the number of deaths that were attributable to BM was similar in patients who were diagnosed with BM in the platinum-resistant and platinum-sensitive settings—3 of 5 (60.0%) and 6 of 13 (46.2%), respectively (*p* = 1.00).

Other factors such as family history of breast or ovarian cancer (*p* = 1.00), high grade serous carcinoma histology (*p* = 1.00) and FIGO stage (*p* = 1.00) were also not associated with platinum sensitivity at the time of BM diagnosis (Table 4).

**Table 4:** Association of clinical characteristics and platinum sensitivity

Characteristic	Platinum-sensitivity	Platinum-resistance	p*
	Frequency (%)	Frequency (%)	
Family History of Breast or Ovarian Cancer			
No	10 (55.6)	8 (44.4)	1.000
Yes	3 (60.0)	2 (40.0)	
Histology			
HGCS	10 (52.6)	9 (47.4)	1.000
Other histologies	2 (66.7)	1 (33.3)	
FIGO stage			
I-III	11 (55.0)	9 (45.0)	1.000
IV	2 (50.0)	2 (50.0)	
Residual disease after primary surgery			
< 1 cm	12 (66.7)	6 (33.3)	0.014
≥ 1 cm	0 (0)	5 (100)	
Disease progression outside CNS			
No	8 (57.1)	6 (42.9)	1.000
Yes	6 (54.5)	5 (45.5)	
Number of metastasis			
1	6 (66.7)	3 (33.3)	0.423
> 1	7 (46.7)	8 (53.3)	
Largest size of brain metastasis			
< 3.2 cm (median)	5 (50.0)	5 (50.0)	0.650
≥ 3.2 cm (median)	6 (66.7)	3 (33.3)	
Number of previous lines of CT			
< 3	11 (78.6)	3 (21.4)	0.017
s≥ 3	3 (27.3)	8 (72.7)	
Death directly related to BM			
No	2 (40.0)	3 (60.0)	1.000
Yes	7 (53.8)	6 (46.2)	

*HGSC* High grade serous carcinoma, *CNS* Central nervous system, *BM* Brain metastasis *p values calculated with Exact Fisher’s test

## Discussion

BM from ovarian cancer is a rare but severe event in the course of the disease. In this study, of 560 ovarian cancer patients, we evaluated data on 26 patients who developed BM. Their overall survival was poor, with a median overall survival of 10.8 months, and 72% of them died due to the BM. Performance status, a diagnosis of BM in the platinum-resistant setting, the time from the initial diagnosis to that of BM, and the number of previous treatment lines correlated with shorter survival. Notably, the number and size of BMs and the presence of disease outside of the central nervous system were unrelated to survival.

A poor median overall survival from the diagnosis of BM has been observed in other series. The 2 largest previous series and 1 systematic review reported a median overall survival of 6.2 months, [13] 8.2 months, [3] and 12.0 months, [15] respectively. The impact of BM on survival could be underestimated if it is mistaken for a late event in patients toward the end of life who die from other mechanisms that are related to systemic disease. In our study, 72.2% of deaths were directly attributable to BM, and 57.4% did not have concurrent disease progression outside of the central nervous system; these rates did not differ between patients with platinum-sensitive and platinum-resistant recurrence. An earlier study that evaluated the prognostic impact of various sites of recurrence in ovarian cancer showed that patients with BMs have the worst prognosis [21]. Collectively, these data suggest that BM is not an indirect marker of late-stage disease but is instead a direct mechanism of death in most ovarian cancer patients with BM.

Platinum-free interval is one of the most important prognostic factors in the recurrence of ovarian cancer [22]. Most previous series of patients with BM from ovarian cancer did not consider this specific characteristic of ovarian cancer patients. Sehouli et al. screened 4077 ovarian cancer patients to find 74 patients with BM and showed that a diagnosis of BM in the platinum-sensitive setting was associated with better survival, with an HR of 0.23 [13]. Liu et al. analyzed 29 patients and also noted a correlation between platinum sensitivity and overall survival, wherein patients who experienced platinum-resistant recurrence had a 5.13-fold greater chance of death [16]. The more recent and larger MITO study evaluated platinum sensitivity as a prognostic marker and found no link between platinum sensitivity and the prognosis [15]. However, it defined platinum sensitivity based on the platinum-free interval of the first recurrence, whereas our study, Sehouli et al., and Liu et al. classified platinum sensitivity, based on the platinum-free interval at the time of the diagnosis of the BM [13, 16]. Among published series these three studies were the only ones that reported the prognostic impact of PFI in the moment of BM diagnosis, and the last one from the MITO group used a different definition of platinum sensitivity. With our study we add data on the importance of PFI in the decision-making process in this scenario.

A higher number of previous treatment lines and a longer interval since the primary diagnosis to the diagnosis of BM were associated with a shorter survival. These findings highlight the importance of the behavior of the disease with regard to the prognosis, because patients with more treatment lines and a longer period since the primary diagnosis are at greater risk of platinum-resistant recurrence. In our study, patients with 3 or more previous treatment lines had a higher likelihood of platinum-resistant recurrence. Although the time from the primary diagnosis has been evaluated in other series, with varying results, [17, 18] the number of previous treatment lines has not been examined.

Among the classical prognostic factors for survival in patients with BM [5, 6], only performance status was relevant in our study. Age, disease progression outside of the central nervous system, and the number and size of BMs have been linked to overall survival in previous studies [3, 13–15, 18] but were unrelated to overall survival in our cohort. None of these factors was related to platinum sensitivity at the time of BM, arguing against platinum sensitivity as a confounding factor.

We did not have data on *BRCA* status for our patients, but 7 studies have reported these data for 37 patients with BM. Among them, 21 patients carried a pathogenic *BRCA* mutation [13, 23–25]. There are no data for ovarian cancer patients with BM regarding the prognostic impact of *BRCA* mutations. Disease-specific prognostic scores for breast [7] and lung cancer [26] have already incorporated molecular subtypes and driver mutations. This deficiency is a limitation of our and previous reports on ovarian cancer and should be considered in future studies.

Other study limitations are due primarily to the small number of patients, limiting the multivariate analysis what does not allow us to completely exclude the possibility of confounding factors leading to the results seen in univariate analysis, and generating low statistical power in excluding the importance of prognostic factors that are otherwise not associated with survival. However, considering the rarity of BM in ovarian cancer patients, this series has yielded significant data, recognizing the factors that are specific to ovarian cancer patients, such as platinum sensitivity and multiple lines of treatment.

## Conclusions

In conclusion, the factors that are related to platinum sensitivity and BM as an early event during the course of the disease appear to be more related to survival than the classical factors that are usually associated with survival in BM from other cancers.

## Data Availability

The datasets used and/or analyzed during the current study are available from the corresponding author on reasonable request.

## Abbreviations

BM: Brain Metastasis
CNS: Central nervous system
DS-GPA: Disease-Specific Grade Prognostic Index
ECOG: Eastern Cooperative Oncology Group
GCIG: Gynecological Cancer Intergroup
HR: Hazard ratio
IQR: Interquartile range
KPS: Karnofsky performance status
OS: Overall survival
PFI: Platinum-free interval
RECIST: Response Evaluation Criteria in Solid Tumors
RT: Radiotherapy
